# Sinus Node Dysfunction after Successful Atrial Flutter Ablation during Follow-Up: Clinical Characteristics and Predictors

**DOI:** 10.3390/jcm11113212

**Published:** 2022-06-04

**Authors:** Guan-Yi Li, Fa-Po Chung, Tze-Fan Chao, Yenn-Jiang Lin, Shih-Lin Chang, Li-Wei Lo, Yu-Feng Hu, Ta-Chuan Tuan, Jo-Nan Liao, Ting-Yung Chang, Ling Kuo, Cheng-I Wu, Chih-Min Liu, Shin-Huei Liu, Wen-Han Cheng, Shih-Ann Chen

**Affiliations:** 1 Division of Cardiology, Department of Medicine, Taipei Veterans General Hospital, Taipei 11217, Taiwan; lgy8065@gmail.com (G.-Y.L.); eyckeyck@gmail.com (T.-F.C.); linyennjiang@gmail.com (Y.-J.L.); ep.slchang@msa.hinet.net (S.-L.C.); gyrus1975@gmail.com (L.-W.L.); huhuhu0609@gmail.com (Y.-F.H.); duan.dachuan@gmail.com (T.-C.T.); care1980@gmail.com (J.-N.L.); tingyungchang@gmail.com (T.-Y.C.); kl19860209@gmail.com (L.K.); shawnwu64@gmail.com (C.-I.W.); sasuke9301108@hotmail.com (C.-M.L.); shinhueiliu0101@gmail.com (S.-H.L.); hill55772003@hotmail.com (W.-H.C.); epsachen@ms41.hinet.net (S.-A.C.); 2 Department of Medicine, School of Medicine, National Yang Ming Chiao Tung University, Taipei 112304, Taiwan; 3 Cardiovascular Center, Taichung Veterans General Hospital, Taichung 40705, Taiwan

**Keywords:** atrial flutter, catheter ablation, permanent pacemaker, sinus node dysfunction, temporary pacemaker

## Abstract

Identification of sinus node dysfunction (SND) before termination of persistent AFL by catheter ablation (CA) is challenging. This study aimed to investigate the characteristics and predictors of acute and delayed SND after AFL ablation. We retrospectively enrolled 221 patients undergoing CA of persistent AFL in a tertiary referral center. Patients with SND who required a temporary pacemaker (TPM) after termination of AFL or a permanent pacemaker (PPM) during follow-up were identified. Acute SND requiring a TPM was found in 14 of 221 (6.3%) patients following successful termination of AFL. A total of 10 of the 14 patients (71.4%) recovered from acute SND. An additional 11 (5%) patients presenting with delayed SND required a PPM during follow-up, including 4 patients recovering from acute SND. Of these, 9 of these 11 patients (81.8%) underwent PPM implantation within 1 year after the ablation. In multivariable analysis, female gender and a history of hypothyroidism were associated with the requirement for a TPM following termination of persistent AFL, while older age and a history of hypothyroidism predicted PPM implantation. This study concluded that the majority of patients with acute SND still require a PPM implantation despite the initial improvement. Therefore, it is reasonable to monitor the patients closely for at least one year after AFL ablation.

## 1. Introduction

Atrial flutter (AFL), a common atrial tachyarrhythmia, includes both typical and atypical forms. Radiofrequency catheter ablation (CA) has been implemented to terminate AFL with promising results [1]. However, sinus node dysfunction (SND) may coexist in patients with AFL. The presence of SND can become notable after termination of AFL, and a pacemaker may be required [2,3]. Detection of SND before termination of AFL is clinically challenging. At present, there are limited studies investigating the predictors or risk factors for SND following termination of AFL.

Sinus node inactivity caused by atrial tachyarrhythmias might be reversed following successful CA, and then the acute SND could recover. However, even without acute SND or after the recovery of SND following AFL ablation, sinus node function may still deteriorate in some patients during follow-up. It is a dilemma to determine the duration of observation in patients with acute SND after termination of persistent AFL and the exact time for permanent pacemaker (PPM) implantation. 

Therefore, our study aimed to examine clinical characteristics and predictors of acute and delayed SND after successful elimination of persistent AFL, including typical and atypical forms.

## 2. Materials and Methods

### 2.1. Study Population

We retrospectively enrolled patients with persistent AFL, including typical and/or atypical AFL, who underwent CA at Taipei Veterans General Hospital between January 2014 and January 2020. The “persistent” AFL was defined as tachycardia lasting for more than 7 days, whilst long-standing persistent AFL was defined as sustained AFL for more than 12 months. Patients who had previously received AFL ablation or had a PPM implantation before the AFL ablation were excluded. Meanwhile, patients who had previously undergone ablation for other types of arrhythmias were still eligible. The Institutional Review Board of Taipei Veterans General Hospital approved this study in accordance with Good Clinical Practice Guidelines.

### 2.2. Baseline Patient Characteristics

Patients’ demographics and preprocedural comorbidities are included in the database, such as age, body mass index, coronary artery disease, valvular heart disease, hypertension, diabetes mellitus, heart failure with reduced ejection fraction, chronic kidney disease, history of transient ischemic attack or stroke, hyperthyroidism, hypothyroidism, and atrial fibrillation. Additionally, the pharmacological history one year before and after the ablation, including beta-blockers, non-dihydropyridine calcium channel blockers, propafenone, and amiodarone, was documented.

The pre-procedural echocardiography was reviewed, and we recorded parameters including left atrial (LA) and right atrial (RA) diameter, LA and RA area, left ventricular ejection fraction, left ventricular hypertrophy, as well as moderate and severe mitral/tricuspid regurgitation. The minor-axis RA diameter, LA, and RA area were all measured by the 4-chamber view at end-systole according to recommendations from the American Society of Echocardiography. The 12-lead electrocardiography and ambulatory electrocardiography monitoring records within one year before the ablation were reviewed. If neither of these records revealed a sinus rhythm, the patient was classified with a “long-standing persistent AFL”. Otherwise, the last recorded heart rate in sinus rhythm would be identified. Furthermore, the QRS duration prior to ablation was analyzed.

### 2.3. Electrophysiological Study and Catheter Ablation

Each patient signed an informed consent form. A standard electrophysiological study and CA were conducted for AFL. A decapolar catheter with an interelectrode spacing of 2-5-2 mm was inserted into the coronary sinus, with the proximal bipole located at the ostium. In our study, typical AFL was defined by the cavotricuspid isthmus (CTI) dependence and was confirmed if concealed entrainment was identified when pacing the CTI and if the difference between post-pacing interval at the CTI and flutter cycle length was within 30 ms. The other AFLs would be classified as an atypical form. Before CA, the flutter cycle length was measured at the proximal coronary sinus.

We have previously described in detail the electrophysiological study, mapping, and ablation strategies for AFL [4]. Ablation of the CTI was performed for persistent typical flutter, while linear ablation of the isthmus, which was identified by either 3D electroanatomic activation mapping or entrainment, was used for atypical flutter. Pulmonary vein isolation and/or other ablation strategies were based on patients’ clinical presentation and physicians’ discretion. Conduction block in both directions was confirmed simultaneously with the ablation line. In our laboratory, formal sinus node function testing was not routinely performed during AFL ablation.

### 2.4. Post-Ablation Follow-Up and Pacemaker Implantation

In the present study, we routinely arranged a 12-lead ECG 2 weeks after ablation at the outpatient clinic and the 24-h Holter monitoring 3 months later. If the patient was symptomatic, additional ECG, 24-h Holter monitoring, or a 7-day event recorder was arranged on a case-by-case basis to detect the recurrence of arrhythmias or post-ablation conduction system disorder.

SND is defined as a persistent condition associated with at least one of the following: (1) pronounced sinus bradycardia with a heart rate of fewer than 50 beats per minute, (2) junctional bradycardia, or (3) repeated sinus pauses longer than 3 s, (4) hemodynamic instability or symptoms related to bradycardia or sinus pause, and (5) sinus node recovery time (SNRT) > 1500 ms or corrected sinus node recovery time (CSNRT) > 550 ms.

In our study, we defined an acute SND as one that occurred following the elimination of AFL or in the same hospitalization. A new or recurrent SND during follow-up in an outpatient clinic was classified as a “delayed SND”. Patients receiving either a temporary pacemaker (TPM) or a PPM implantation for SND after AFL ablation were identified. In these cases, we explored the duration of TPM back-up, the time interval between the ablation and the PPM implantation, the pacing modes of the PPM, and the averaged percentage of atrial pacing during follow-up.

### 2.5. Statistical Analysis

The normally distributed continuous variables are presented as means ± standard deviations, and the non-normally distributed continuous variables are presented as medians with 25 and 75% interquartile ranges (IQRs). Wilcoxon signed-rank test (Mann–Whitney U test) or Student’s T-test was used to compare the differences between groups. The categorical variables are expressed as numbers and percentages and compared using the Chi-square test. A *p*-value of <0.05 was considered statistically significant. In this study, logistic regression analysis was used to determine the association between variables and acute SND that required a TPM, and Cox regression analysis was used to determine the predictors of delayed SND that required a PPM implantation after successful AFL ablation during long-term follow-up. The parameters with a *p*-value < 0.05 in the univariable regression analysis were selected for the multivariable model. A Kaplan–Meier survival curve was plotted to determine event-free survival, with the statistical significance examined using the Log-rank test. The statistical analyses were conducted using IBM Corporation’s Statistical Package for the Social Sciences version 22.0 (Armonk, NY, USA).

## 3. Results

### 3.1. Patient Selection, Characteristics of Atrial Flutter, and Catheter Ablation

A total of 245 patients underwent AFL ablation in our tertiary referral center during the study period. After excluding 24 patients, 221 patients were included in the study. As shown in Supplementary Tables S1 and S2, all patients had clinically documented AFL for more than 7 days, and 104 (47.1%) patients had a long-standing persistent AFL. A total of 103 patients (46.6%) with concomitant clinically documented AF were enrolled. A total of 168 (76.0%) patients took at least 1 antiarrhythmic drug before ablation. In our electrophysiological laboratory, 177 (80.1%) patients were diagnosed with counterclockwise typical AFL, 22 (10.0%) patients with clockwise typical flutter, and 55 (24.9%) patients with atypical AFL. Regarding the ablation procedure, CTI ablation, pulmonary vein isolation, peri-superior vena cava (SVC) ablation, and bi-atrial ablation were performed in 204, 21, 5, and 23 patients, respectively. After a median follow-up period of 5.0 months (25–75% IQR: 0.7–6.5 months), AFL recurred in 19 patients (8.6%). A total of 6 patients (2.7%) received a repeat procedure of AFL ablation.

### 3.2. Clinical Characteristics of Sinus Node Dysfunction

**Figure 1:**
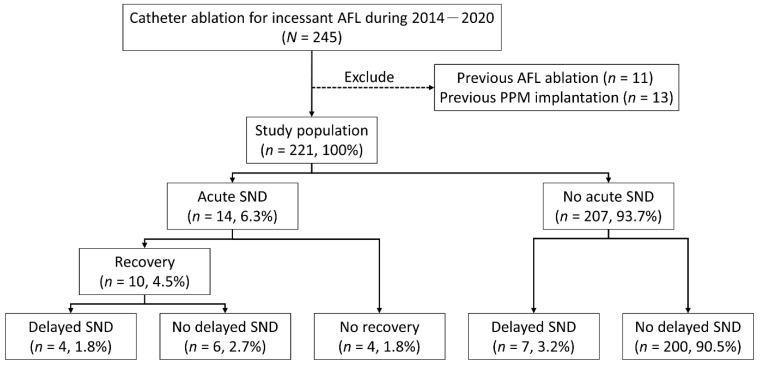
Flow chart of the studied patients. AFL: atrial flutter; PPM: permanent pacemaker; SND: sinus node dysfunction.

Acute SND requiring a TPM was identified in 14 (6.3%) patients following termination of AFL (Figure 1). Additionally, 4 patients (28.6%) had unrecoverable SND and required implantation of a PPM 3.5 days (25–75% IQR: 0.8–4.0 days) after ablation. For the other 10 patients who recovered from the acute SND, all TPMs were removed successfully within 5 days, and in the majority (8 out of 10, 80%) within 2 days (Table 1). After a median follow-up period of 4.2 months (25–75% IQR: 3.9–8.0 months), 4 of the 14 patients (28.6%) developed delayed SND. In total, 8 of the 14 patients (57.1%) with acute SND underwent implantation of a PPM. Before catheter ablation for the 14 patients with acute SND, dizziness, fatigue, and syncope were documented in 4, 1, and 1 patient(s), respectively. Among these 6 patients, the acute SND was resolved in 4 patients. However, 2 of them developed subsequent delayed SND requiring a PPM implantation. For the remaining 207 (93.7%) patients without acute SND, delayed SND requiring a PPM was found in 7 (3.4%) patients after a median follow-up period of 4.7 months (25–75% IQR: 1.6–11.9 months) (Figure 1).

**Figure 2:**
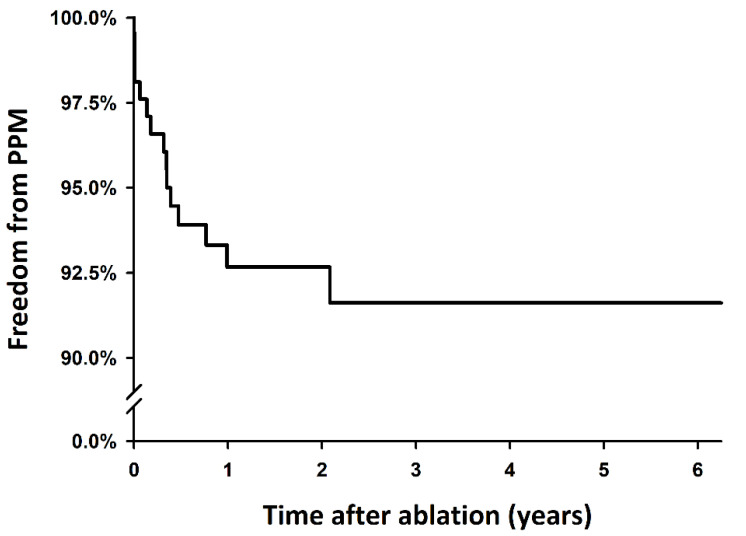
Kaplan–Meier estimates for the PPM implantation after ablation for persistent AFL. After ablation for persistent AFL, the cumulative incidence of delayed SND requiring a PPM implantation increased for several months, almost reaching a plateau after 1 year. AFL: atrial flutter; PPM: permanent pacemaker; SND: sinus node dysfunction.

In the 11 patients exhibiting delayed SND, most (9 out of 11, 81.8%) of the PPMs were implanted within one year following the ablation. Figure 2 shows the PPM-free survival curve after the ablation. After ablation, the cumulative incidence of delayed SND requiring a PPM implantation increased for several months after CA, almost reaching a plateau after 1 year. A total of 12 of the 15 patients underwent dual chamber mode (DDD) PPM implantation, while the remaining 3 underwent single chamber mode (VVI) PPM implantation. All the 8 PPMs implanted for patients with acute SND were in the DDD mode (Table 1). After the PPM implantation, 7 patients suffered from paroxysmal AF, and 3 had AFL recurrences. One patient underwent a repeat procedure for AFL ablation. For the 12 PPMs with DDD mode, after 2 weeks, 3 months, and 1 year following the implantation, the average percentage of atrial pacing was 58.2%, 56.0%, and 58.4%, respectively.

### 3.3. Predictors of Sinus Node Dysfunction

The baseline characteristics of patients with and without acute SND requiring a TPM are summarized in Supplementary Table S1, and those with and without delayed SND requiring a PPM are presented in Supplementary Table S2.

Based on the univariable logistic regression analysis, a female gender, a history of hypothyroidism, and an increased LA diameter assessed by transthoracic echocardiography were found to be significant predictors of acute SND requiring a TPM implantation after termination of AFL. The multivariable stepwise models showed that female gender and hypothyroidism were the independent predictors [*p* = 0.038, OR: 3.66, 95% confidence interval (CI): 1.08–12.43; *p* = 0.045, OR: 8.80, 95% CI: 1.05–74.03, respectively] (Table 2).

**Figure 3:**
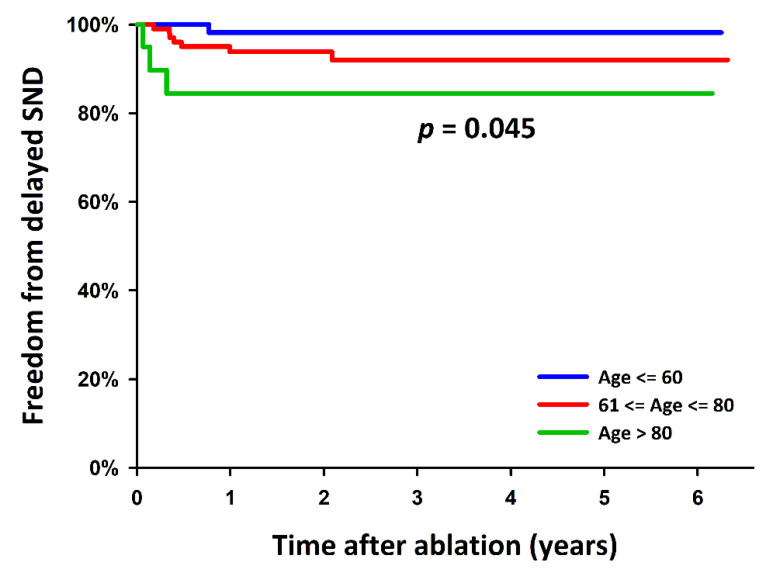
Kaplan–Meier estimates for the delayed SND requiring a PPM after ablation for persistent AFL among patients in the different age groups, with the statistical significance examined using the Log-rank test. AFL: atrial flutter; PPM: permanent pacemaker; SND: sinus node dysfunction.

**Figure 4:**
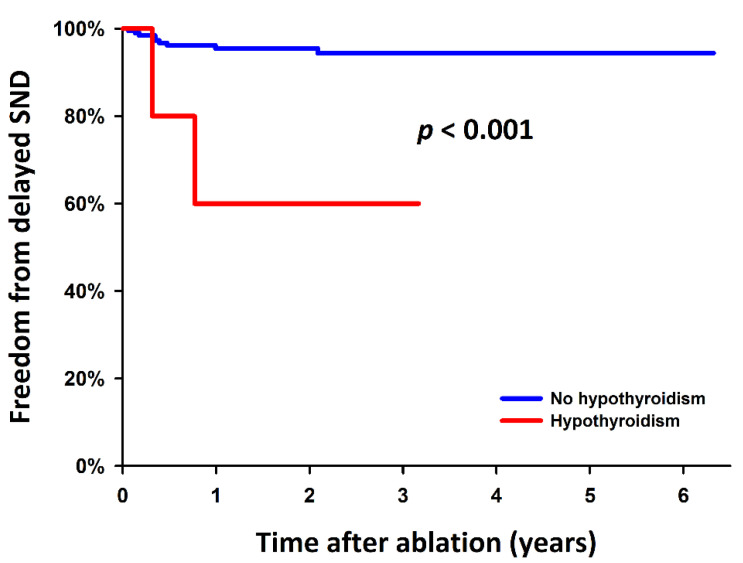
Kaplan–Meier estimates for the delayed SND requiring a PPM after ablation for persistent AFL among patients with and without a history of hypothyroidism, with the statistical significance examined using the Log-rank test. AFL: atrial flutter; PPM: permanent pacemaker; SND: sinus node dysfunction.

Moreover, as a result of the univariable Cox proportional hazards regression analysis, older age and a history of hypothyroidism were significant predictors of delayed SND requiring a PPM implantation after AFL termination. In the stepwise multivariable model, both factors were independent predictors (*p* = 0.018, HR: 1.07, 95% CI: 1.01–1.13; *p* = 0.006, HR: 8.87, 95% CI: 1.89–41.72, respectively) (Table 3). Patients with older age and a history of hypothyroidism had higher rates of delayed SND requiring a PPM implantation during the follow-up after AFL ablation (Log-rank test, *p* = 0.045 and <0.001, respectively; Figure 3 and Figure 4).

## 4. Discussion

### 4.1. Major Findings

After successful elimination of the AFL, 14 (6.3%) patients required a TPM for acute SND. It should be noted that although some of them initially recovered from acute SND, 57.1% of patients eventually required a PPM. Irrespective of the absence or recovery of acute SND, 11 (5.0%) patients developed delayed SND requiring a PPM during follow-up, mostly within 1 year after ablation (9 out of 11, 81.8%). The underlying history of hypothyroidism and older age were both predictors of delayed SND.

### 4.2. The Mechanism of Sinus Node Dysfunction following Atrial Flutter Elimination

SND, historically referred to as sick sinus syndrome, is commonly caused by senescence of the sinoatrial node and surrounding atrial muscle. Most frequently, it is associated with atrial arrhythmias as part of the tachycardia–bradycardia syndrome (TBS) [2,3]. The mechanisms of TBS include alterations in myocardial architecture, ion channel metabolism, and gene expression [5,6,7]. Over the past decade, catheter ablation has advanced significantly, enabling a high proportion of AF and AFL to be completely eliminated [8,9,10]. Typically, SND will become apparent after atrial arrhythmias have been eliminated. 

The second possible mechanism of SND following CA is an iatrogenic injury to the sinus node. During peri-SVC ablation for AF or AFL, direct damage to the sinus node has been reported, especially along the anterolateral free wall of the SVC [11,12]. Additionally, if the sinoatrial nodal artery is the only artery that supplies the sinus node, an injury to this artery may result in SND [13]. Given the anatomic consideration, occlusion of the sinoatrial nodal artery during LA anterior line ablation of perimetral flutter may result in SND [14,15]. Ozturk et al. found that ablation near the medial or posterior aspects of the LA appendage could also damage the sinoatrial nodal artery [16].

Some other studies, however, provided contradictory results. Deshmukh et al. demonstrated that the risk of SND after ablation is similar to cardioversion in patients with AF, suggesting that causality may be due to a common electrophysiologic substrate as opposed to the ablation process itself [17]. The present study provided evidence in support of these findings by demonstrating that no ablation site for AFL could significantly predict subsequent SND (Table 2 and Table 3). In addition, as shown in the supplementary Tables S1 and S2, 5 of 221 patients in our cohort underwent peri-SVC ablation, and none of them developed acute or delayed SND.

### 4.3. Characteristics of Acute and Delayed Sinus Node Dysfunction

SND has been reported to be reversed after the elimination of atrial tachyarrhythmias. It has been demonstrated in an animal model by Raitt MH et al. that electrical remodeling can be reversed following termination of persistent AF by cardioversion [18]. Several human studies have also demonstrated a reverse remodeling of sinus node function following CA for AF [7,19,20,21]. In contrast, Inada et al. found that SND progressed even after paroxysmal AF had been eliminated after a 5-year follow-up [22]. The discrepancy between these findings implies that acute SND observed after termination of AFL could recover in some patients, whereas sinus node function may still deteriorate during follow-up in patients without acute SND or after the recovery of the initial SND. Due to the variability and uncertainty of sinus node function following elimination of AFL, it is clinically challenging to determine the appropriate duration for observation and the time point for advancing to PPM implantation.

In the retrospective study by Song et al., 8% of patients developed acute SND after AFL ablation, but half of these cases were transient, and most of them improved within a day [23]. Furthermore, Semmler et al. reported that acute SND developed in 40 (3.2%) patients undergoing AF ablation. There were 37.5% of patients with acute SND of a transient nature, and all of their TPMs were removed within 2 days [24]. Similarly, in our study, 6.3% of patients had acute SND after AFL ablation. TPM was successfully removed in most of these cases within two days, except for one case on the third day and another case on the fifth day after ablation. Accordingly, for the recovery from SND, a reasonable period for observation would be around 1 week.

According to Song et al., the median time for PPM implantation after AFL ablation was 20.5 days (25–75% IQR: 15.25 to 38.25 days), although 1 patient had PPM implantation 1 year and 7 months after CA [23]. In Kim et al.’s study, 121 patients underwent CA for AF with TBS, and 11 patients (9.1%) received PPM within a median period of 21 months after CA [25]. There was a wide variation between the times of ablation and the times for PPM implantation. Moreover, we observed in the present study that most PPMs for delayed SND were implanted several months after AFL ablation and gradually plateaued after 1 year. It is a reasonable and safe policy to closely monitor for delayed SND for at least one year after AFL ablation, especially in patients with a high risk of SND.

Skjøth F et al. found that PPM was implanted more frequently following AFL ablation than AF ablation in a nationwide cohort study [26]. As indicated above, this finding appears to be consistent with a comparison of the present study with those mentioned. Furthermore, patients with AFL tend to experience a shorter interval between catheter ablation and PPM implantation than those with AF. Possible explanations for the above findings may be related to the different remodeling of the heart. Medi C et al. reported that right atrial remodeling was more advanced in patients with AFL as compared with patients with AF [27]. It may lead to a higher degree of SND and the early requirement of PPM after AFL ablation.

### 4.4. Predictors of Sinus Node Dysfunction following Atrial Flutter Termination

In previous studies, several risk factors or predictors of SND after termination of AF have been reported, including older age, a female gender, a low preprocedural ventricular rate, and a large left atrium [24,28,29,30,31,32]. The present study found that age, female gender, and the underlying history of hypothyroidism were predictive of SND after AFL ablation. SND is associated with senescence of the sinoatrial node and surrounding atrial myocardium, and this phenomenon is frequently accompanied by the process of aging. As demonstrated both in the present study as well as in previous studies, older age can serve as a predictor for SND following termination of atrial tachyarrhythmias [17,24,28]. Moreover, Sairaku et al. reported that female gender was an independent predictor of SND requiring a PPM in cases of persistent typical flutter [33], and higher rates of SND and PPM implantation have also been reported after AF ablation in female patients [17,28], which was consistent with our findings. 

Thyroid hormones are both inotropic and chronotropic by multiple mechanisms, including the regulation of the sympathetic nervous system and ion channels [34]. For the comprehensive investigation of the relationship between thyroid hormone deficiency and SND, we checked the medical history and laboratory data of the 5 patients with hypothyroidism in our cohort. All these 5 patients had been under thyroxine supplements, but 2 of them developed delayed SND after recovery from acute SND (Supplementary Tables S1 and S2). The thyroid function test showed euthyroid in 1 case but still hypothyroid in the other during the peri- and post-ablation period before PPM implantation. Thyroxine deficiency might not be the only contributor to the SND in hypothyroidism, and it requires further studies to determine the reversibility of cardiac remodeling and sinus node function after thyroxine supplementation. Despite the identified risk factors, given the small number of patients with SND following AFL termination, future validation is necessary.

### 4.5. Strengths and Limitations

This is, to the best of our knowledge, the first analysis of the predictors, characteristics, and prognosis of acute and delayed SND following ablation for persistent AFL, including typical and atypical forms, as well as the clinical application of TPM and PPM.

Nevertheless, there are some limitations to this study. The retrospective design of this study results in some inevitable biases. Additionally, we excluded patients with severe atrioventricular nodal dysfunction, which might have confounded the primary endpoint of SND. Furthermore, the cause of SND may be multifactorial, and it is difficult to clearly clarify the etiology in most of our patients. A previous study used computed tomography to assess the post-ablation sinus nodal artery injury [13], but we did not perform it routinely for our cohort. Thus, it is difficult to evaluate the confounding effect of the causes of SND on the outcomes. Finally, the sample size was small, and the follow-up period was short. Further large-scale studies are needed to determine the long-term incidence and prognosis of SND. We, however, believe that the current findings can not only optimize the pre-procedural risk stratification but also serve as guidance for short-term and long-term management for patients with SND following ablation.

## 5. Conclusions

The TPM was required for acute SND in 6.3% of patients after the elimination of the AFL. Despite initially recovering from acute SND, 57.1% of patients with acute SND eventually required a PPM. It should also be noted that irrespective of the absence or recovery of acute SND, 5.0% of patients developed delayed SND requiring a PPM during follow-up, mostly within 1 year after ablation. An observation period of at least one year after AFL ablation is reasonable.

## Figures and Tables

**Table 1:** The detailed characteristics of the patients developing acute SND after the AFL ablation.

Patient No.	Age (Year)	Gender	AFL Form	Flutter Cycle Length (ms)	Pre-Ablation Sinus Rate (bpm) ^†^	TPM Back-Up Duration (Day)	PPM Mode
Patients who did not recover from acute SND							
1	62	F	Typical	296	NA	3 ^‡^	DDD
2	64	M	Both	348	NA	4 ^‡^	DDD
3	43	F	Typical	294	NA	4 ^‡^	DDD
4	54	M	Typical	264	NA	1 ^‡^	DDD
Patients who recovered from acute SND, but developed delayed SND							
5	60	F	Atypical	208	65	2	DDD
6	61	M	Typical	220	NA	1	DDD
7	78	F	Typical	244	NA	1	DDD
8	89	M	Typical	300	NA	5	DDD
Patients who recovered from acute SND, without developing delayed SND							
9	56	F	Both	288	NA	1	NA
10	68	F	Typical	309	78	3	NA
11	61	F	Typical	238	NA	2	NA
12	62	F	Typical	278	108	1	NA
13	82	F	Atypical	286	64	1	NA
14	89	M	Both	209	NA	1	NA

^†^ Pre-ablation sinus rate will not be available if no documented sinus rhythm during one year before ablation; ^‡^ The TPMs were not removed until PPM implantation; AFL: atrial flutter; bpm: beats per minute; F: female; M: male; NA: not applicable; No.: number; PPM: permanent pacemaker; SND: sinus node dysfunction; TPM: temporary pacemaker.

**Table 2:** Logistic regression analysis of variables to predict acute SND requiring a TPM after AFL termination (*n* = 221).

Variables	Univariable		Multivariable	
	OR (95% CI)	*p* Value	OR (95% CI)	*p* Value
Age	1.02 (0.97–1.06)	0.504		
Female	5.10 (1.64–15.89)	0.005	3.66 (1.08–12.43)	0.038
BMI	0.96 (0.84–1.11)	0.597		
Comorbidities				
CAD	1.05 (0.32–3.49)	0.933		
MR ^†^	1.22 (0.37–4.07)	0.742		
Hypertension	1.48 (0.50–4.43)	0.480		
Diabetes mellitus	1.11 (0.33–3.67)	0.870		
HFrEF	1.79 (0.57–5.60)	0.316		
Chronic kidney disease	1.56 (0.33–7.46)	0.579		
TIA/stroke	1.91 (0.22–16.48)	0.555		
Hyperthyroidism	2.49 (0.50–12.30)	0.264		
Hypothyroidism	11.33 (1.73–74.39)	0.011	8.80 (1.05–74.03)	0.045
AFL type				
CCW typical flutter	1.10 (0.30–4.14)	0.883		
CW typical flutter	1.47 (0.18–11.79)	0.718		
Atypical flutter	1.74 (0.56–5.45)	0.338		
Location of flutter circuit(s)				
Right atrium alone	1.24 (0.15–10.02)	0.841		
Left atrium alone	0.85 (0.18–3.96)	0.834		
Both atriums	0.92 (0.11–7.48)	0.936		
Ablation site(s)				
CTI	2.13 (0.44–10.43)	0.349		
PVI	1.39 (0.17–11.19)	0.757		
Biatrial ablation	1.55 (0.19–12.39)	0.682		
Flutter cycle length	1.01 (0.99–1.01)	0.210		
Concomitant AF	1.57 (0.53–4.69)	0.417		
Pre-procedural medication				
Beta-blocker	1.75 (0.57–5.40)	0.331		
Non-DHP CCB	1.04 (0.34–3.23)	0.944		
Propafenone	0.98 (0.21–4.62)	0.983		
Amiodarone	1.92 (0.65–5.68)	0.241		
Post-procedural medication				
Beta-blocker	0.33 (0.10–1.07)	0.065		
Non-DHP CCB	1.01 (0.27–3.78)	0.988		
Propafenone	1.21 (0.32–4.56)	0.775		
Amiodarone	0.92 (0.31–2.74)	0.880		
Echocardiography				
LA diameter	1.08 (1.01–1.15)	0.031	1.07 (0.99–1.15)	0.062
LA area	1.02 (0.94–1.11)	0.685		
RA diameter	1.05 (0.99–1.13)	0.123		
RA area	1.07 (0.99–1.16)	0.093		
LVEF	0.99 (0.95–1.04)	0.739		
LVH	0.56 (0.12–2.60)	0.458		
MR ^†^	1.06 (0.23–4.97)	0.942		
TR ^†^	1.37 (0.36–5.19)	0.641		
Electrocardiography				
Heart rate ^‡^	1.01 (0.96–1.07)	0.612		
Long-standing persistent AFL	3.01 (0.91–9.89)	0.070		
QRS duration	1.01 (0.98–1.03)	0.598		

^†^ Defined as moderate to severe regurgitation; ^‡^ Only measured for patients with documented sinus rhythm within one year before ablation (*n* = 114); AAD: antiarrhythmic drugs; AF: atrial fibrillation; AFL: atrial flutter; BMI: body mass index; CAD, coronary artery disease; CCB: calcium channel blocker; CCW: counterclockwise; CTI: cavotricuspid isthmus; CW: clockwise; DHP: dihydropyridine; HFrEF: heart failure with reduced ejection fraction; LA: left atrium; LVEF: left ventricular ejection fraction; LVH: left ventricular hypertrophy; MR: mitral regurgitation; OR: odds ratio; PVI: pulmonary vein isolation; RA: right atrium; SND: sinus node dysfunction; TIA: transient ischemic stroke; TPM: temporary pacemaker; TR: tricuspid regurgitation.

**Table 3:** Cox regression analysis of variables to predict delayed SND requiring a PPM after AFL termination (*n* = 221).

Variables	Univariable		Multivariable	
	HR (95% CI)	*p* Value	HR (95% CI)	*p* Value
Age	1.07 (1.01–1.12)	0.016	1.07 (1.01–1.13)	0.018
Female	1.44 (0.42–4.91)	0.564		
BMI	0.97 (0.83–1.12)	0.665		
Comorbidities				
CAD	1.52 (0.45–5.20)	0.503		
MR ^†^	1.25 (0.33–4.73)	0.740		
Hypertension	1.94 (0.57–6.61)	0.292		
Diabetes mellitus	0.27 (0.03–2.10)	0.211		
HFrEF	2.89 (0.88–9.47)	0.080		
Hyperthyroidism	1.13 (0.15–8.83)	0.907		
Hypothyroidism	9.48 (2.04–44.09)	0.004	8.87 (1.89–41.72)	0.006
AFL type				
CCW typical flutter	0.41 (0.05–3.23)	0.400		
Atypical flutter	1.68 (0.49–5.73)	0.411		
Location of flutter circuit(s)				
Right atrium alone	1.00 (0.13–7.81)	0.998		
Left atrium alone	1.10 (0.24–5.09)	0.907		
Both atriums	1.18 (0.15–9.25)	0.874		
Ablation site(s)				
CTI	1.11 (0.14–8.69)	0.920		
PVI	1.07 (0.14–8.34)	0.951		
Flutter cycle length	1.00 (0.98–1.01)	0.670		
Concomitant AF	1.37 (0.42–4.49)	0.604		
Pre-procedural medication				
Beta-blocker	0.89 (0.27–2.91)	0.843		
Non-DHP CCB	0.90 (0.26–3.08)	0.868		
Propafenone	0.50 (0.06–3.92)	0.511		
Amiodarone	0.94 (0.28–3.22)	0.926		
Post-procedural medication				
Beta-blocker	0.41 (0.12–1.39)	0.151		
Non-DHP CCB	0.73 (0.16–3.38)	0.687		
Propafenone	1.38 (0.37–5.21)	0.632		
Amiodarone	0.41 (0.11–1.55)	0.190		
Echocardiography				
LA diameter	1.04 (0.96–1.13)	0.358		
LA area	1.05 (0.96–1.16)	0.294		
RA diameter	0.95 (0.87–1.05)	0.320		
RA area	1.02 (0.91–1.14)	0.755		
LVEF	0.99 (0.93–1.05)	0.645		
LVH	1.95 (0.57–6.66)	0.287		
MR ^†^	0.66 (0.14–3.12)	0.603		
TR ^†^	2.39 (0.62–9.26)	0.206		
Electrocardiography				
Heart rate ^‡^	0.96 (0.90–1.03)	0.274		
Sinus bradycardia ^‡,§^	5.94 (0.99–35.57)	0.051		
Long-standing persistent AFL	1.66 (0.51–5.46)	0.401		
QRS duration	0.99 (0.97–1.02)	0.553		

^†^ Defined as moderate to severe regurgitation; ^‡^ Only measured for patients with documented sinus rhythm within one year before ablation (*n* = 114); ^§^ Defined as sinus rate < 60 beats per minute within one year before ablation; AAD: antiarrhythmic drugs; AF: atrial fibrillation; AFL: atrial flutter; BMI: body mass index; CAD, coronary artery disease; CCB: calcium channel blocker; CCW: counterclockwise; CTI: cavotricuspid isthmus; DHP: dihydropyridine; HFrEF: heart failure with reduced ejection fraction; HR: hazard ratio; LA: left atrium; LVEF: left ventricular ejection fraction; LVH: left ventricular hypertrophy; MR: mitral regurgitation; PPM: permanent pacemaker; PVI: pulmonary vein isolation; RA: right atrium; SND: sinus node dysfunction; TR: tricuspid regurgitation.

## Data Availability

The data presented in this study are not publicly available due to upcoming publications but are available on request from the corresponding author.

## Supplementary Materials

The following supporting information can be downloaded at: https://www.mdpi.com/article/10.3390/jcm11113212/s1, Table S1: Comparison of baseline characteristics between patients with and without acute SND requiring a TPM after AFL termination; Table S2: Comparison of baseline characteristics of patients with and without delayed SND requiring a PPM after AFL termination.
