# Supplementary material for: Sinus Node Dysfunction after Successful Atrial Flutter Ablation during Follow-Up: Clinical Characteristics and Predictors
Source: J Clin Med. 2022 Jun 4;11(11):3212. doi: 10.3390/jcm11113212 (PMC9181344; doi:10.3390/jcm11113212)
Supplement: Supplementary file 1 [file jcm-11-03212-s001.zip › jcm-1749374-supplementary.pdf]

Supplementary Table S1. Comparison of baseline characteristics between patients with and without acute SND requiring a TPM after AFL termination (n = 221)

Variables	No acute SND (n=207)	Acute SND (n=14)	P value
**Age**	63.9±12.9	67.3±13.5	0.362
**Female**	54 (26.1%)	9 (64.3%)	0.004
**BMI**	25.6±4.1	25.0±5.0	0.591
**Comorbidities**			
CAD	57 (27.5%)	4 (28.6%)	0.999
MR†	51 (24.6%)	4 (28.6%)	0.753
Hypertension	98 (47.3%)	8 (57.1%)	0.477
Diabetes mellitus	55 (26.6%)	4 (28.6%)	0.999
HFrEF	49 (23.7%)	5 (35.7%)	0.338
Chronic kidney disease	20 (9.7%)	2 (14.3%)	0.636
TIA/stroke	8 (3.9%)	1 (7.1%)	0.451
Hyperthyroidism	13 (6.3%)	2 (14.3%)	0.244
Hypothyroidism	3 (1.4%)	2 (14.3%)	0.033
**AFL type**			
CCW typical flutter	166 (80.2%)	11 (78.6%)	0.999
CW typical flutter	21 (10.1%)	1 (7.1%)	0.999
Atypical flutter	50 (24.2%)	5 (35.7%)	0.345
**Location of flutter circuit(s)**			
Right atrium alone	189 (91.3%)	13 (92.9%)	0.999
Left atrium alone	34 (16.4%)	2 (14.3%)	0.999
Both atriums	16 (7.7%)	1 (7.1%)	0.999
**Ablation site(s)**			
CTI	192 (92.8%)	12 (85.7%)	0.293
PVI	20 (9.7%)	1 (7.1%)	0.999
Peri-SVC	5 (2.4%)	0 (0.0%)	0.999
Biatrial ablation	22 (10.6%)	1 (7.1%)	0.999
**Flutter cycle length**	256.7±54.4	279.2±40.6	0.179
**Concomitant AF**	95 (45.9%)	8 (57.1.5%)	0.414
**Pre-procedural medication**			
Beta-blocker	105 (50.7%)	9 (64.3%)	0.326
Non-DHP CCB	72 (34.8%)	5 (35.7%)	0.999
Propafenone	30 (14.5%)	2 (14.3%)	0.999
Amiodarone	71 (34.3%)	7 (50.0%)	0.257
**Post-procedural medication**			
Beta-blocker	114 (55.1%)	4 (28.6%)	0.054
Non-DHP CCB	44 (21.3%)	3 (21.4%)	0.999
Propafenone	38 (18.4%)	3 (21.4%)	0.727
Amiodarone	93 (44.9%)	6 (42.9%)	0.880
**Echocardiography**			
LA diameter	42.7±7.9	48.3±6.7	0.018
LA area	20.9±6.5	21.6±6.8	0.687
RA diameter	34.4±7.4	37.8±12.0	0.320
RA area	16.7±5.3	19.6±9.5	0.302
LVEF	52.4±11.6	51.3±7.3	0.738
LVH	134 (77.0%)	12 (85.7%)	0.739
MR†	30 (15.0%)	2 (14.3%)	0.999
TR†	33 (16.6%)	3 (21.4%)	0.710
**Electrocardiography**			
Heart rate‡	74.1±17.6	78.8±20.5	0.615

Sinus bradycardia‡§		24 (11.6%)	0 (0.0%)	0.578
Long-standing persistent	AFL	94 (45.4%)	10 (71.4%)	0.059
QRS duration		105.8±22.6	108.1±26.5	0.738

† Defined as moderate to severe regurgitation; ‡ Only measured for patients with documented sinus rhythm within one year before ablation (n=114); § Defined as sinus rate < 60 bpm within one year before ablation; AAD: antiarrhythmic drugs; AF: atrial fibrillation; AFL: atrial flutter; BMI: body mass index; CAD: coronary artery disease; CCB: calcium channel blocker; CCW: counterclockwise; CTI: cavotricuspid isthmus; CW: clockwise; DHP: dihydropyridine; HFrEF: heart failure with reduced ejection fraction; LA: left atrium; LVEF: left ventricular ejection fraction; LVH: left ventricular hypertrophy; MR: mitral regurgitation; PVI: pulmonary vein isolation; RA: right atrium; SND: sinus node dysfunction; SVC: superior vena cava; TIA: transient ischemic stroke; TPM: temporary pacemaker; TR: tricuspid regurgitation.

Supplementary Table S2. Comparison of baseline characteristics of patients with and without delayed SND requiring a PPM after AFL termination (n = 221)

**Variables**	**No delayed SND (n=210)**	**Delayed SND (n=11)**	**P value**
**Age**	63.8±12.9	68.9±12.8	0.142
**Female**	59 (28.1%)	4 (36.4%)	0.513
**BMI**	25.6±4.2	25.3±3.4	0.761
**Comorbidities**			
CAD	57 (27.1%)	4 (36.4%)	0.501
MR†	52 (24.8%)	3 (27.3%)	0.999
Hypertension	99 (47.1%)	7 (63.6%)	0.286
Diabetes mellitus	58 (27.6%)	1 (9.1%)	0.296
HFrEF	49 (23.3%)	5 (45.5%)	0.142
Chronic kidney disease	22 (10.5%)	0 (0.0%)	0.607
TIA/stroke	9 (4.3%)	0 (0.0%)	0.999
Hyperthyroidism	14 (6.7%)	1 (9.1%)	0.547
Hypothyroidism	3 (1.4%)	2 (18.2%)	0.021
**AFL type**			
CCW typical flutter	167 (79.5%)	10 (90.9%)	0.697
CW typical flutter	22 (10.5%)	0 (0.0%)	0.607
Atypical flutter	51 (24.3%)	4 (36.4%)	0.473
**Location of flutter circuit(s)**			
Right atrium alone	192 (91.4%)	10 (90.9%)	0.999
Left atrium alone	34 (16.2%)	2 (18.2%)	0.696
Both atriums	16 (7.6%)	1 (9.1%)	0.594
**Ablation site(s)**			
CTI	194 (92.4%)	10 (90.9%)	0.594
PVI	20 (9.5%)	1 (9.1%)	0.999
Peri-SVC	5 (2.4%)	0 (0.0%)	0.999
Biatrial ablation	23 (11.0%)	0 (0.0%)	0.610
**Flutter cycle length**	257.4±54.5	265.4±45.2	0.610
**Concomitant AF**	97 (46.2%)	6 (54.5%)	0.588
**Pre-procedural medication**			
Beta-blocker	108 (51.4%)	6 (54.5%)	0.840
Non-DHP CCB	73 (34.8%)	4 (36.4%)	0.999
Propafenone	31 (14.8%)	1 (9.1%)	0.999
Amiodarone	74 (35.2%)	4 (36.4%)	0.999
**Post-procedural medication**			
Beta-blocker	114 (54.3%)	4 (36.4%)	0.245
Non-DHP CCB	45 (21.4%)	2 (18.2%)	0.999
Propafenone	38 (18.1%)	3 (27.3%)	0.432
Amiodarone	96 (45.7%)	3 (27.3%)	0.353
**Echocardiography**			

LA diameter	43.0±8.0	43.9±6.2	0.727
LA area	20.8±6.5	23.2±4.9	0.316
RA diameter	34.7±7.9	31.8±5.3	0.290
RA area	16.9±5.8	17.5±3.5	0.779
LVEF	52.4±11.5	50.4±9.2	0.558
LVH	139 (78.5%)	7 (63.6%)	0.268
MR†	30 (14.7%)	2 (20.0%)	0.648
TR†	33 (16.3%)	3 (30.0%)	0.378
**Electrocardiography**			
Heart rate‡	74.7±17.9	65.6±19.6	0.271
Sinus bradycardia‡§	21 (10.0%)	3 (27.3%)	0.061
Long-standing persistent AFL	98 (46.7%)	6 (54.5%)	0.610
QRS duration	106.3±23.2	100.9±15.5	0.388

† Defined as moderate to severe regurgitation; ‡ Only measured for patients with documented sinus rhythm within one year before ablation (n=114); § Defined as heart rate < 60 bpm within one year before ablation; AAD: antiarrhythmic drugs; AF: atrial fibrillation; AFL: atrial flutter; BMI: body mass index; CAD: coronary artery disease; CCB: calcium channel blocker; CCW: counterclockwise; CTI: cavotricuspid isthmus; CW: clockwise; DHP: dihydropyridine; HFrEF: heart failure with reduced ejection fraction; LA: left atrium; LVEF: left ventricular ejection fraction; LVH: left ventricular hypertrophy; MR: mitral regurgitation; PPM: permanent pacemaker; PVI: pulmonary vein isolation; RA: right atrium; SND: sinus node dysfunction; SVC: superior vena cava; TIA: transient ischemic stroke; TR: tricuspid regurgitation.
